# Gamma irradiation effects on physical and electronic properties of monolayer CVD grown graphene†

**DOI:** 10.1039/d5na00162e

**Published:** 2025-06-03

**Authors:** Chintan P. Chavda, Ashok Srivastava, Erin Vaughan, Jianwei Wang, Manas Ranjan Gartia, Georgios Veronis

**Affiliations:** a Division of Electrical and Computer Engineering, Louisiana State University Baton Rouge LA USA cchavd2@lsu.edu; b United States Airforce Research Laboratory Albuquerque NM USA erin.vaughan.1@us.af.mil; c Department of Mechanical and Industrial Engineering, Louisiana State University Baton Rouge LA USA mgartia@lsu.edu; d Center for Computation and Technology, Louisiana State University Baton Rouge LA USA gveronis@lsu.edu; e Department of Geology and Geophysics, Louisiana State University Baton Rouge LA USA jianwei@lsu.edu; f Department of Mechanical Engineering, The Pennsylvania State University, University Park Pennsylvania 16803 USA

## Abstract

Two-dimensional material graphene has proven to have remarkable electrical and photonic properties, opening the door to a wide range of uses, including employment under the harsh conditions of space. The creation of graphene on various substrate types is known to be possible *via* a number of approaches, including direct deposition and the substrate transfer process. In this work, we used an argon plasma, methane as a carbon source, and a nanoCVD-8G graphene reactor to deposit monolayer graphene (MLG) on transition metal substrates for studying the effects of gamma irradiation on the physical and electronic properties. Graphene's crystalline structure is investigated utilizing Raman and X-ray Photo Electron Spectroscopy (XPS) techniques before and after gamma irradiation. The results show that point defects predominate in the damage following gamma irradiation. The defective structure and electronic properties are connected in light of density functional theory (DFT) simulations of pristine and defective graphene.

## Abstract 2

We describe the effects of gamma irradiation on CVD produced graphene that was deposited on transition metal substrates.

## Graphical abstract

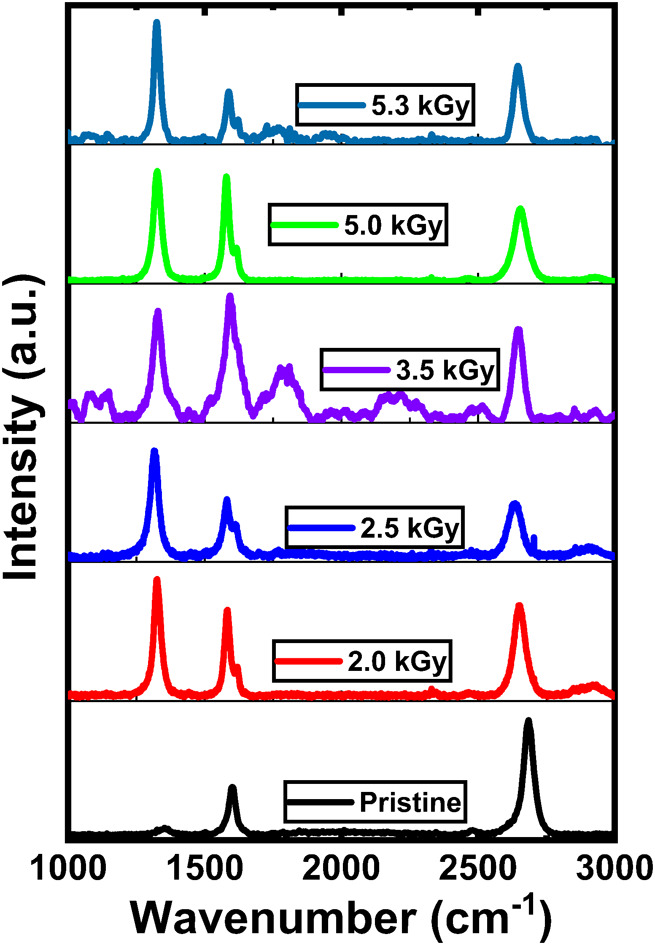

## Introduction

Graphene, a two-dimensional material,^1^ has exceptional electronic and photonic properties that make it suitable for a wide range of applications.^2–9^ According to Moore's law the number of transistors doubles every 16 to 19 months. As Moore's law suggests, the transistor technology has scaled rapidly and reached the level where the channel length of silicon transistors is currently very small.^10^ As a result, there is a very high leakage current in very small silicon technologies.^11^ Therefore, there is a need for novel materials to overcome this issue. Graphene has promising electronic properties for very small technologies and is a promising option for future technology. However, transmission electron spectroscopy, scanning electron microscopy, Raman spectroscopy, and X-ray photo-electron diffraction techniques are needed to manufacture graphene-based devices and analyse graphene. In particular, in space electronics, where harsh environmental factors such as temperature, X-rays, alpha particles, beta particles and gamma radiation sources are present, these can create single event effects and influence the reliability of electronic devices.^12^ The atomic structure of the crystal lattice of graphene can be damaged, disordered, and subject to faults as a result of such hostile conditions.^13^ It is therefore vital to investigate how irradiation affects the crystalline structure and electronic properties of graphene.

Recent developments have extended the advances in graphene far beyond its original electronic applications. Graphene and its derivatives have been used as supports for catalysis. Recent work demonstrated non-covalent functionalization routes for metal nanoparticle integration and selective hydrogenation reactions.^14^ In addition, graphene quantum dots, nanoribbons, and frameworks have been engineered for enhanced performance in heterogeneous photocatalysis, owing to their tunable electronic and surface properties.^15^ A broader perspective on these advancements, as well as the future role of graphene in energy and photocatalytic technologies, is detailed in a review by Zhang *et al.*, which emphasizes the integration of graphene in next-generation composite systems for solar fuel generation and pollutant degradation.^16^

Recent studies have been focused on the effect of e-beams and ion irradiation including swift heavy ion irradiation on the properties of graphene and graphene devices.^17–28^ Childres *et al.* investigated the effects of e-beam irradiation on graphene field effect transistors (GFETs).^17^ Iqbal *et al.* studied e-beam irradiation effects on CVD grown graphene.^18^ Compagnini *et al.* investigated ion irradiation on MLG.^19^ Kalbac *et al.* performed studies of ion irradiation induced defects on two-layered graphene.^20^ Ochedowski *et al.* studied irradiation hardness of graphene and MoS_2_ field effect devices against swift heavy ion irradiation.^21^ Iqbal *et al.* investigated e-beam irradiation effects on CVD grown graphene.^23^ Akcöltekin *et al.* studied effects of swift heavy ions on graphene.^24^ Mathew *et al.* performed studies on effects of mega-electron-volt proton irradiation on graphene.^25^ Lehtinen *et al.* studied effects of ion irradiation on graphene.^26^ Zhang *et al.* investigated ionizing dose effects on graphene based non-volatile memory devices.^27^ Alexandrou *et al.* studied the improvement in radiation hardness of GFETs.^28,29^ However, studies of the effects of gamma irradiation on graphene materials are limited with a focus on multilayer graphene and graphene oxide over insulator substrates.^22^ A metallic substrate is expected to greatly affect the physical properties and irradiation response of graphene materials, especially monolayer graphene, compared to an insulator substrate. E-beam and ion irradiation methods often result in localized lattice damage, sputtering, and thermal effects due to direct particle interactions with the graphene surface.^26,28^ In contrast, gamma irradiation provides uniform, non-contact exposure without inducing mechanical or thermal disruption.^30^

In this article, we describe the effects of gamma irradiation on CVD produced graphene that was deposited on transition metal substrates. Raman spectroscopy and XPS were employed for the characterization of the irradiation effect and electronic structure calculations based on density function theory (DFT) were used to facilitate the interpretation and understanding of the observations.

Monolayer graphene was created on transition metal substrates using a graphene nano-CVD reactor. Using Raman and X-ray photo-electron spectroscopy, we investigated the flaws and electrical behavior of irradiated graphene. We conducted Raman spectroscopy research, in contrast to the majority of the published literature, without moving the graphene to a SiO_2_/Si substrate. The transfer procedure frequently uses lithography, etching, and lift-off methods, which can cause the graphene layer to become wrinkled and imperfect and increase contaminants. For these reasons, we have undertaken a study investigating the effects of gamma irradiation on graphene deposited on a metallic substrate. For our experiment, we used a ^60^Co source for the irradiation, which has a nominal irradiation dose rate of 2.07 Gy min^−1^.

## Materials and methods

The monolayers of CVD-grown graphene over transition metal substrates were deposited using the CVD process. The ^60^Co (cobalt-60) source at the Louisiana State University Nuclear Science Department was used to irradiate the materials. The source has a nominal radiation dose rate of 1.91 Gy min^−1^ (±5%). The total number of samples was *n* = 5, and samples were assigned a number # 1–5. The gamma irradiation dose was 2.00 kGy, 2.5 kGy, 3.00 kGy, 5.00 kGy and 5.30 kGy for samples # 1, 2, 3, 4 and 5, respectively. After these irradiation doses, we performed Raman spectroscopy and XPS studies.

### Monolayer graphene deposition (CVD process)

We deposited a monolayer of graphene using Moorfield Nanotechnology's nanoCVD-8G reactor by the CVD process. CVD is a widely used technology to produce high quality graphene over a large area of transition metal (Cu, Ru, Pb and Ni) substrates using hydrocarbon gases.^31^ Hydrocarbon gases (methane and ethylene) are introduced in the chamber which decompose over the metallic substrate at high temperature. The metal works as a catalyst in this process. As the temperature decreases, the solubility of the carbon atoms decreases, and the carbon atoms form the intended film in two dimensions so that graphene is formed. In Fig. S1† we showed the schematic of a cold wall resistive heater type CVD system. Five different substrates of transition metals (two Ni and three Cu) were used for deposition of the monolayer of graphene. The size of substrates was 1 cm × 1 cm. First, the substrates were cleaned using isopropyl alcohol. With the carbon precursor, CH_4_ = 10%, H_2_ = 5%, and Ar = 85% for 120 seconds at a chamber pressure of 10 torr at 1000 °C, a monolayer graphene film was deposited over the transition metal substrates.

### Gamma irradiation setup

Chavda *et al.* showed the gamma irradiation setup for the study of the effects of gamma irradiation on the physical properties of MoS_2_ monolayers.^30^ We use a dry irradiator with a ^60^Co source to irradiate the samples. Decay-corrected dose rates were calculated to determine the required irradiation time for the different samples. All samples were placed at the same position in the irradiator chamber to ensure geometrical uniformity. The samples were placed five inches from the source based on the manufacturer's recommendations for the irradiator. The dose rate was measured to be 2.07 Gy min^−1^ (207 rad min^−1^). As our ^60^Co source has a nominal dose rate of 2.07 Gy min^−1^ (207 rad min^−1^) (±5%), sample 1 was irradiated for 17.02 (8.51 + 8.51) hours to achieve an irradiation of 2.0 kGy, sample 2 was irradiated for 42.5 (21.25 + 21.25) hours to achieve 2.5 kGy, and sample 3 was irradiated for 10.63 + 10.63 hours to achieve 1.25 kGy. Sample 4 was irradiated for 14.89 hours to achieve 1.75 kGy and sample 5 was irradiated for 22.54 hours to achieve 2.65 kGy irradiation of gamma rays.

### Raman spectroscopy

We used a Renishaw inVia Reflex Raman spectroscope for the Raman experiments. We used a laser excitation wavelength of 632 nm in all experiments. We used a 100× objective lens and a 10-second acquisition time to get the Raman spectra. WiRE 5.3, software used exclusively for the examination of Raman spectra, was used to analyse the collected spectra. We used the extended mode. We analysed its peaks using the OriginPro software suite.

The D, G, and 2D peaks of the crystalline monolayer graphene were examined using Raman spectroscopy. The D peak was especially employed to search for structural flaws (defects) in graphene. By examining the G peak, the 2D peak, and the intensity ratio of the D peak to the G peak, the transition in the crystalline structure of graphene was discovered.

### X-ray photoelectron spectroscopy

A Scienta Omicron ESCA 2SR X-ray photoelectron spectroscope outfitted with a Mg/Al monochromatic source was used to conduct X-ray photoelectron spectroscopy (XPS) studies. The XPS data were analysed using the CASA XPS software suite. Each stage of the experiment included sample analysis using X-ray Photo-electron Spectroscopy (XPS). Four peaks were identified in the XPS data at energies of 284.8 eV, 285.3 eV, 286.0 eV, and 288.5 eV, which, respectively, corresponded to the C–C, C–OH, C–O–C, and COOH bonds. We considered the area covered by different bonds in the XPS data at each stage of the experiment to study the electrical conductivity of graphene.

### DFT calculations

We performed computational investigations using the DFT approach implemented using the Quantum ESPRESSO Suite.^32,33^ The primary objective was to investigate the electronic properties of both pristine and irradiated monolayers of graphene. By utilizing this computational tool (Quantum Espresso suite), we obtained important insights into the effects of irradiation on the electronic structure of graphene.

The Kohn–Sham equation is solved using the Quantum ESPRESSO suite to determine a system's electrical characteristics. The Quantum ESPRESSO suite can identify the band structure and density of states using the solution of the Kohn–Sham equation. A 5 × 5 supercell was developed for the computation using the BURAI software suite, which is a graphical user interface for Quantum ESPRESSO. The generalized gradient approximation (GGA) was based on exchange–correlation with the Perdew–Burke–Ernzerhof functional (Blöchl, 1994; Perdew *et al.*, 1992).^34,35^ Due to its ability to balance accuracy and computing efficiency, the GGA approach is frequently chosen for materials research and chemical simulations. In order to account for non-uniform electron distributions, which are vital for bonding patterns, the GGA integrates information on the electron density gradient. The cutoff energy of 50 Ry, the kinetic energy for the plane-wave basis, was stopped. A pristine MLG structure with 50 atoms, a 5 × 5 cell size, 200 electrons, and 120 Kohn–Sham states was investigated with Quantum ESPRESSO computations. The K points 12 12 12 0 0 0 and 1.00000 × 10^−8^ were used as the convergence threshold. Optimization was done using the relax technique, which only allows for atomic location variation. The convergence threshold (conv. thr.) is the maximum permissible change in the total energy between two consecutive rounds of the self-consistent field (SCF) cycle. The *k* points are used to sample the Brillouin zone and to calculate integrals over the reciprocal lattice vectors. The system was stable, and the optimization converged inside the set threshold, according to the results. USPP-type (ultra-soft pseudopotential) pseudopotentials from the Quantum ESPRESSO website's PS Library were used for the computation. USPPs model the ionic core using a smooth, soft pseudopotential that rapidly decays away from the nucleus, providing a more flexible description of the valence electrons. Unlike typical pseudopotentials, which have an abrupt cutoff, this pseudopotential has a seamless transition between the core and valence electrons. One advantage of USPP is the larger plane-wave basis set, which is more flexible and provides more accurate representations of the electrical structure. This results in more accurate estimates of total energy and charge density, especially in systems with complex bonds or those where relativistic effects are important.

## Results and discussion

### Raman spectrum results

**Fig. 1:**
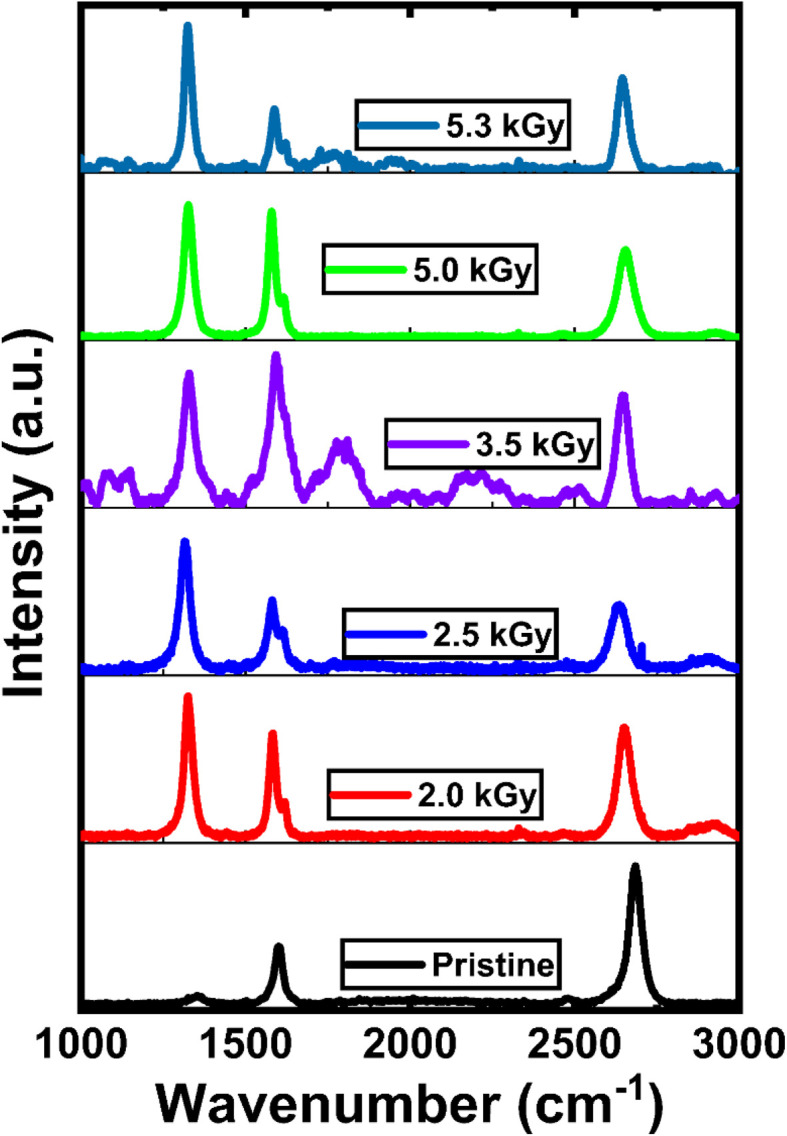
Raman spectrum results of monolayer graphene after different gamma irradiation doses.

Graphene's Raman spectra exhibit a D peak at around 1350 cm^−1^, a G peak at about 1580 cm^−1^, and a 2D peak at about 2700 per cm wavenumber.^36^ The in-plane vibration of sp^2^ carbon atoms corresponds to the zone center phonons of *E*_2g_ symmetry that are linked to the G peak. The graphene layer count can be found from the G and 2D peaks.^37^ The monolayer nature of the graphene is confirmed by the sharp, symmetric 2D peak, which is well-fitted by a single Lorentzian function with no splitting. The *I*_2D_/*I*_G_ intensity ratio of ∼2.4 and a G peak full width at half maximum (FWHM) of 31.15 cm^−1^ are consistent with previously reported values for monolayer graphene.^36,38–40^ The crystal lattice's in-plane optical phonons cause the D peak.^41^ The activation of the D peak in the Raman spectra of graphene indicates defects.^41^ The *k*-point phonons of monolayer graphene's *A*_1g_ symmetry are represented by the D peak.^40^ The D peak in graphene's Raman spectra is also known as the “disorder peak”. The breathing modes of six-atom rings are associated with this peak, which needs defects to be activated. In pristine graphene, the D peak is essentially nonexistent (Fig. 1). There is a substantial correlation between the type and quantity of disorder in the graphene lattice and the intensity and position of the D peak. When gamma radiation was introduced, the Raman spectra of monolayer graphene showed the appearance of the D peak.

Fig. 1 shows the position of the G peak and the 2D peak as a function of gamma irradiation dose. The positions of the G peak and 2D peak shift towards lower wavenumbers (redshift) with respect to the G peak and 2D peak in the Raman spectra of pristine graphene after gamma irradiation (2.0 kGy) was introduced. Gamma irradiation induces point defects into graphene, which can alter its vibrational characteristics, most notably generating a redshift in the Raman spectra.^38^ These shifts of the G peak and 2D peak towards a lower wavenumber after the introduction of gamma irradiation might be due to strain-induced phonon softening due to modification of bond lengths and angles by point defect creation in monolayer graphene.^38,42^ The periodicity and symmetry of the lattice are broken when defects are added to graphene. The position of this G peak can be affected by changes in the graphene structure, such as those brought on by the emergence of defects. The G band is composed of the in-plane vibration of sp^2^ linked carbon atoms. The blueshift of the G band in irradiated graphene has been interpreted by Ferrari *et al.* as a sign of compressive strain.^43^ Localized distortions of the graphene lattice that result in an overall compressive strain can be caused by the insertion of groups containing oxygen or the development of defects such as vacancies.^37,44,45^

The substrate may potentially have an impact on the graphene Raman signals. Charge transfer to graphene can be induced by a metallic substrate, producing a doping effect.^46^ This alters graphene's electrical structure and may cause the Raman peaks to change. SiO_2_ substrates, in contrast, can produce a very different result.^47^ Due to the thermal expansion mismatch with graphene, they frequently cause strain.^48^ They may also trap charges, resulting in accidental doping. These effects are often less pronounced than those brought about by a metallic substrate.^44,49^

After further irradiation doses, the G peak moves towards a higher wavenumber, which indicates an ordering exactly opposite to that of the graphene (crystalline structure), which may be due to the amorphization of graphene.^18^ The blueshift (shift towards a higher wavenumber) of 2D bands may be due to hole doping by creating point defects in the crystalline structure of monolayer graphene.^41^ The blueshift is suggestive of phonon hardening too. This blueshift might result from amorphization, or the change from a crystalline to a disordered state.^50^ Vibrational frequencies may arise as a result of the disruption of the hexagonal carbon lattice in graphene in its amorphous state, which increases disorder and causes differences in link lengths and angles.^51^ Furthermore, these vibrational modes may be impacted by localized strains introduced by a larger defect density brought on by enhanced gamma irradiation.^52^

**Fig. 2:**
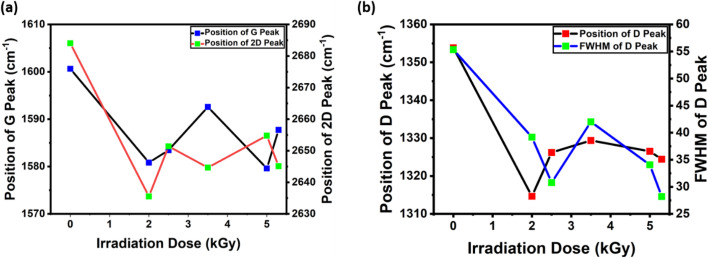
(a) Position of the G peak and 2D peak as a function of different gamma irradiation doses. (b) Position and full width at half maximum (FWHM) of the D peak as a function of gamma irradiation dose.

In Fig. 2(b), we show the position of the D peak and the FWHM of the D peak as a function of gamma irradiation. As we introduced gamma irradiation, the D peak position moved to a lower wavenumber. A redshift in the Raman spectra of graphene's D peak indicates a decrease in vibrational energy associated with phonon modes affected by defects.^40^ This shifting can be attributed to variations in disorder or the nature of introduced defects. The dynamic environment local to the graphene lattice can be changed by adding complex defect configurations or increasing the density of simpler defects. These alterations are frequently seen as a redshift in the D peak, which indicates phonon softening in the vicinity of defect-rich regions.^53^ The redshift (signaling phonon softening) in the Raman peaks is typically associated with tensile strain, and the precise positional change of the D peak may depend on the type of strain (compressive or tensile) and the kinds of defects that are present.^54^ After 2.5 kGy of irradiation, the peak position moves to a slightly higher wavenumber (blueshift) from that under 2.0 kGy of irradiation. This trend continues under 3.5 kGy of irradiation. This result suggests that the vibrations surrounding defects have become “stiffer” in relation to the D peak, which may indicate a reduction in disorder or the healing of defects. Following exposure to irradiation, graphene could go through annealing procedures. A few defects may be “healed” during this, especially those that are not very energetically unfavorable. In order to rebuild a perfect hexagonal lattice, carbon atoms must rearrange throughout this healing process, which lowers the defect density.^55^ As a result, the D peak blueshifts as the defects get fewer and the disorder of the system gets lower. Under certain circumstances, defective graphene may interact chemically with nearby molecules, such as those found in ambient air. This may result in chemical reactions that passivate the defects or heal them partially.^56^ A blueshift in the D peak may result from such chemical interactions or passivation stiffening the vibrational modes associated with defects. The blueshift in the Raman spectra of monolayer graphene indicates a reduction in disorder or the repair of defects. The exact reasons for the blueshift depend on the treatments or environments that the graphene has been exposed to after radiation.^51^ After further gamma irradiation, it again starts to move towards lower wavenumbers (redshift) for 5.0 kGy and 5.3 kGy (redshift) of irradiation.

FWHM of the D peak decreases for the first few initial doses, and then it starts to recover itself, but at higher irradiation doses of 5.0 kGy and 5.3 kGy, FWHM decreases again. The introduction of uniform types of flaws by initial doses of radiation may result in more homogeneous defects.^41^ The reason for the recovery of FWHM is that partial annealing or reorganization of defects may occur at intermediate doses.^51^ The introduction of uniform types of defects by initial doses of radiation may result in more homogeneous defects.

The FWHM amplitude is low where the *I*_D_/*I*_G_ is high (2.0 kGy, 2.5 kGy, 5.0 kGy and 5.3 kGy). An increase in the FWHM of the D band indicates increasing disorder in the graphene structure.^57^ High doses (5.0 kGy and 5.3 kGy) have the potential to generate more complicated or diversified defects, which would increase disorder and decrease FWHM. This is why the FWHM of the D peak decreases again at high doses.^58^ The FWHM amplitude is low where the *I*_D_/*I*_G_ is high (2.0 kGy, 2.5 kGy, 5.0 kGy and 5.3 kGy). This implies that the defects introduced by gamma irradiation are more uniform in nature.^52^

The disorder or crystal structural flaws are linked to the D peak in the Raman spectra of 2-D materials (graphene). The G peak, which denotes the crystallinity of graphene, is associated with the in-plane stretching motion of pairs of sp^2^ bound carbon atoms. The degree of material flaws is frequently assessed using the *I*_D_/*I*_G_ ratio.

**Fig. 3:**
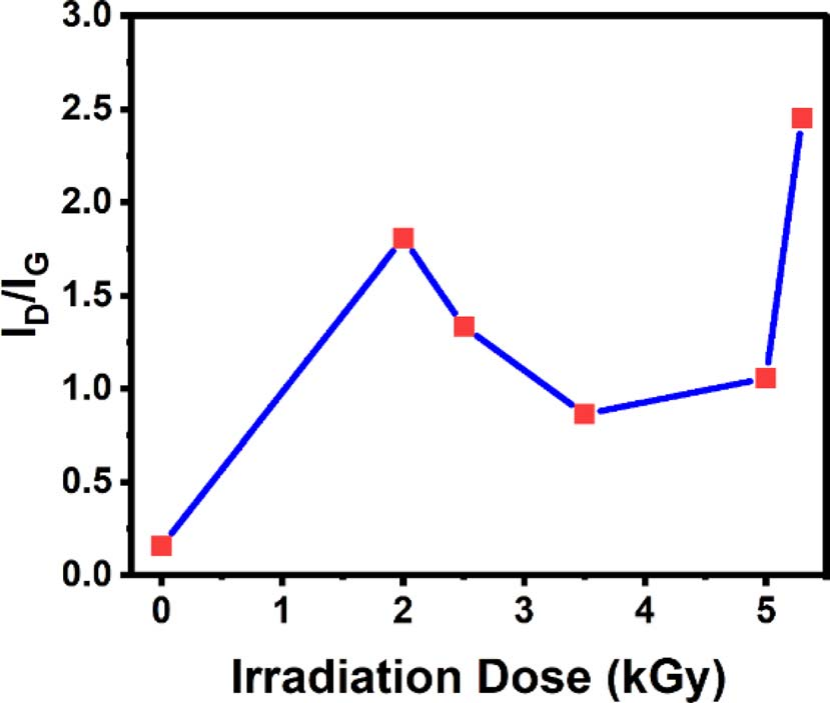
Evolution of *I*_D_/*I*_G_ as a function of gamma irradiation dose.

In Fig. 3, we show the intensity ratio of D and G peaks (*I*_D_/*I*_G_) as a function of gamma irradiation dose. After the first dose of 2.0 kGy, the intensity ratio of the D and G peaks increases and then starts to decrease after 2.5 and 3.5 kGy of irradiation. After 5.0 kGy and 5.3 kGy of irradiation, *I*_D_/*I*_G_ increases. The increase in the *I*_D_/*I*_G_ ratio at low radiation doses (0.0 kGy to 2.0 kGy) suggests the occurrence of flaws or disarray in the carbon network, such as voids or interstitials. These flaws cause the sp^2^ bond to break down, hence enhancing the D peak.^59^ The annealing or “healing” of these flaws may be the reason why the *I*_D_/*I*_G_ ratio begins to decline following larger doses of radiation. The local heating brought on by the strong irradiation can encourage defect recombination and annihilation, restore the sp^2^ network, lower the D peak intensity, and lower the *I*_D_/*I*_G_ ratio.^60^ The first two trends are explained by the graphene to amorphous carbon (amorphization) trajectory.^38^ An increase in *I*_D_/*I*_G_ is due to the change of crystalline graphene into nanocrystalline graphene, and a decrease in *I*_D_/*I*_G_ is associated with the transformation of nanocrystalline graphene into the majority of sp^2^ amorphous carbon.^18^

### X-ray photoelectron spectroscopy (XPS) studies

**Fig. 4:**
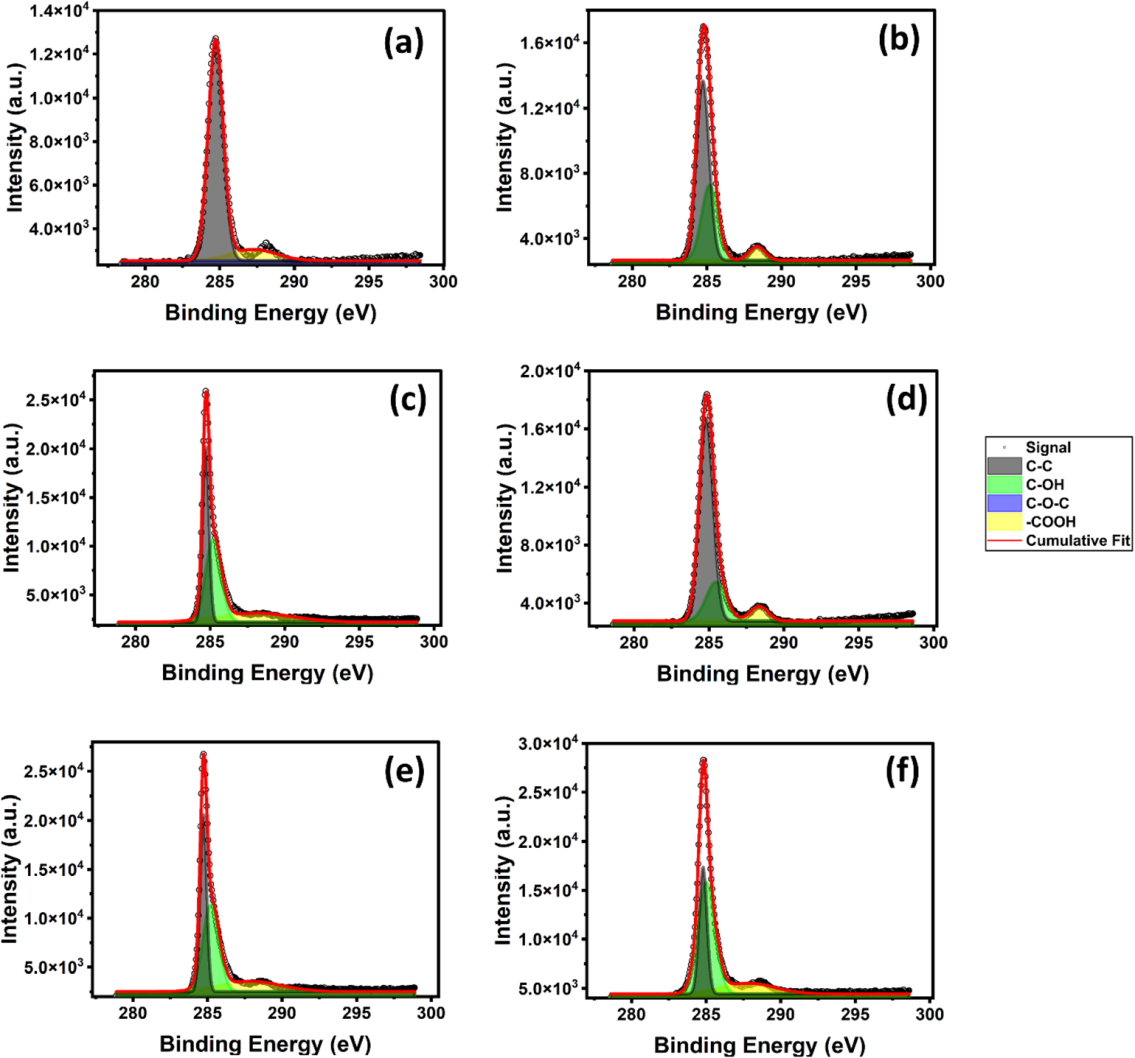
XPS spectra of graphene with (a) 0 kGy; (b) 2.0 kGy; (c) 2.5 kGy; (d) 3.5 kGy; (e) 5.0 kGy; (f) 5.3 kGy of gamma irradiation.

To learn more about the impacts of gamma irradiation on monolayer graphene (MLG), we studied MLG samples using X-ray photoelectron spectroscopy (XPS). In Fig. 4 we have shown the XPS spectra of MLG. We observed the peaks for C–C, C–OH, C–O–C, and COOH bonds, which are positioned at 284.8 eV, 285.3 eV, 286.0 eV, and 288.5 eV binding energies, respectively. The XPS spectra of the pristine and irradiated samples are shown in Fig. 4. Notably, we found that all samples have the largest peak at 284.8 eV binding energy, which is a signature of graphene.^61^ The C 1s peak position is typically centered at 284.8 eV, which is associated with the sp^2^-hybridized carbon atoms in the honeycomb lattice structure of graphene.^40^ Additionally, we noticed a tiny shoulder near the –COOH bond in the pure sample, which was probably brought on by contact with ambient oxygen.^49,52,62,63^

**Fig. 5:**
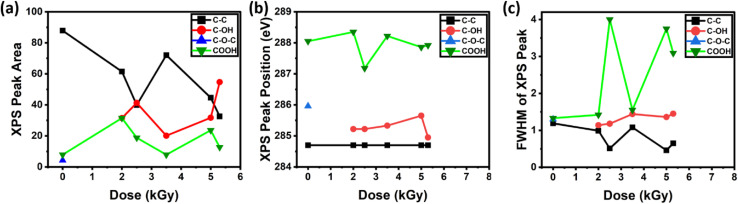
(a) XPS peak area as a function of irradiation dose. (b) XPS peak position as a function of irradiation dose. (c) Full width at half maximum (FWHM) of XPS peaks as a function of irradiation dose.

Fig. 5(a) presents the XPS peak area of different atomic bonds as a function of gamma irradiation dose. After initial doses of gamma irradiation of 2.0 kGy and 2.5 kGy, the XPS peak area associated with the C–C bond has decreased, and for the 3.5 kGy irradiation dose, the XPS C–C bond peak area has increased, and after further irradiation, the main carbon C–C peak area has decreased. Moreover, after observation, the overall C–C peak area decreased. There is no C–OH peak present in the pristine sample of MLG. The C–OH bond was introduced after the introduction of gamma irradiation to MLG. As we increased the gamma irradiation dose, the C–OH and –COOH were increased overall, which suggests that gamma irradiation has increased the adsorption of oxygen in MLG by creating defects.^28^

To further analyse our XPS results, we studied the XPS peak position of MLG as a function of gamma irradiation dose. The peak position of the C–C bond remains the same for all samples, since the carbon atoms in graphene's honeycomb lattice structure are sp^2^-hybridized, and the C 1s peak position is typically centred at around 284.8 eV.^61^ Our XPS study shows that as the irradiation dose increases, the C–OH bond shifts to higher binding energies.^64,65^ Similar to this, Suk *et al.* and Lerf *et al.*^63,66^ discovered that the peak positions of C–OH bonds in irradiated samples also shifted to higher binding energies with an increasing gamma irradiation dose. This shift suggests an increase in bonding interactions involving oxygen atoms, likely due to the formation of additional functional groups upon irradiation. As a result, these oxygen-involving bonds require higher binding energies, highlighting how irradiation-induced defects can alter the electronic environment of carbon atoms in graphene. For some of the irradiated samples (samples 2–4) the –COOH bond shifts to higher binding energies. Due to the strong contact between the oxygen atom and the carbon atom next to the carboxyl group, the peak position of the –COOH bond is anticipated to shift to higher binding energies.^49,64,66^ This interaction lowers the neighbouring carbon atom's electron density and raises the binding energy needed to knock an electron out of the C 1s orbital.^41,49,67^ Overall, the introduction of defects caused the peak locations of the C–OH, C–O–C, and –COOH bonds in graphene to change.

We also performed a study on the FWHM (full width at half maximum) of XPS peaks (Fig. 5(c)). Similar to the peak area study, with the introduction of gamma irradiation, the FWHM of the main carbon, C–C, decreased and other peaks' (C–OH and –COOH) FWHM increased, which also suggests that, as we increase the intensity of gamma irradiation, oxygen adsorption in MLG increases. The initial decrease in the FWHM of the C–C peak followed by a zigzag pattern may be indicative of an initial ordering or annealing effect of the radiation, followed by intermittent damage or changes in the electronic environment.^68^ An increase in the FWHM of the C–OH peak suggests a range of the C–OH bonding environment. This is probably because there are more defects in the system, which creates a wider range of locations for the formation of hydroxyl groups. Because of the dynamic nature of the irradiation process, the zigzag pattern seen in the –COOH peak suggests alternating processes of production and removal or transformation of these functional groups.^55^ Radiation induced defects in graphene can lead to new sites for oxygen-containing functional groups. Furthermore, there may be interaction between graphene and the copper substrate, particularly if there's copper oxidation or there are other interactions during extended radiation exposure.

### Electronic property study using DFT

The band structure and density of states of pristine and defective graphene (to model irradiated graphene) are investigated to understand the effect of irradiation on the electronic properties of irradiated MLG. The results will be used to show the connection between features from the Raman and XPS and defect formation under irradiation.

**Fig. 6:**
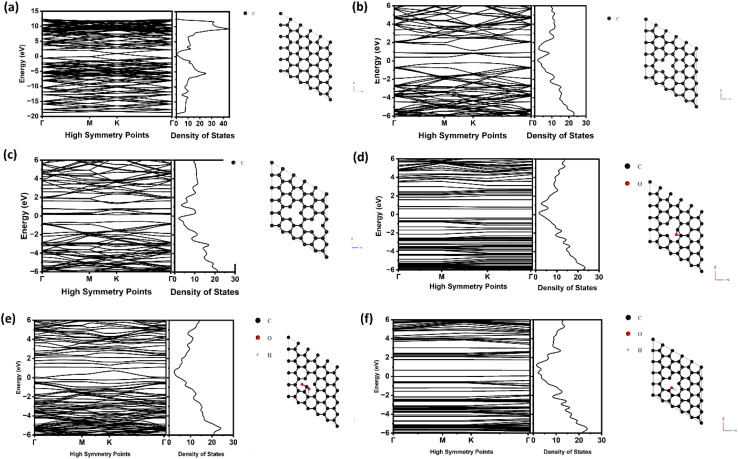
(a) Band structure and density of states of pristine MLG. (b) Band structure and density of states of defective MLG with one point defect. (c) Band structure and density of states of defective MLG with two point defects. (d) Band structure and density of states of MLG with a C–O–C bond impurity. (e) Band structure and density of states of MLG with a –COOH bond impurity. (f) Band structure and density of states of MLG with a C–OH bond impurity.

Fig. 6(a) shows the band structure and density of states of pristine (MLG). At the Dirac point, the density of states is zero, indicating the semi metallic behavior of graphene. However, our experimental studies have shown that exposure to gamma irradiation leads to the formation of point defects, which are dominant in the crystalline structure of MLG. To investigate the effect of such defects on the electronic properties of graphene, we performed DFT calculations on two cases of MLG with one and two defects. Our results indicate a significant shift in the Dirac point, leading to the absence of the Dirac point in both cases, as shown in Fig. 6(b) and (c). The bands are opened and suggest semiconductor character and defect bands occur as inter-bands. These findings suggest that the introduction of point defects through gamma irradiation has a substantial impact on the electronic behavior of graphene, which may have implications for its conductivity and other properties such as reduced conductivity. Bond distances next to the defect sites decrease, causing an increase in bond distance elsewhere. This increase in the bond distance reduces the force constant, leading to redshift of Raman peaks, which is consistent with the vacancy formation mechanism under irradiation.

Apart from the gamma-irradiation-induced point defects, we investigated the impact of particular bond impurities on the electronic structure of MLG which we have observed during our Raman spectra and XPS studies. As shown in Fig. 6(d)–(f), the DFT calculations for MLG with C–O–C, –COOH, and C–OH bond impurities show a noticeable distortion in the band structure and the DOS profiles. Significantly, the introduction of states at the Fermi level by the –COOH and C–OH bond impurities suggests that graphene is changing from a semi-metallic to a semiconducting material. The formation of localized states within the bandgap during this transition dramatically changes the dynamics of charge carriers. These findings are essential for modifying graphene's electrical characteristics for certain uses, such as sensors or transistors, where a controlled bandgap is essential. Our research also sheds light on how resilient graphene's electrical characteristics are to different kinds of atomic-scale disturbances, highlighting graphene's potential use in flexible and durable electronics.

## Conclusions

In conclusion, we investigated accumulative dose effects of gamma irradiation on CVD grown monolayer graphene. We studied the changes in the physical properties of MLG using Raman spectroscopy and XPS. Our results show that point defects are dominant after the gamma irradiation on MLG, which are confirmed using DFT calculations. A D peak forms and gets broader and wider after the introduction of the irradiation dosage and as the irradiation doses are increased; this is a sign that the gamma irradiation of mono-layer graphene has caused defects in MLG. Clear redshift and blueshift occur in the G and 2D peaks in Raman spectra of all samples, which indicates phonon softening by creating defects and bond distance changes as suggested by the DFT calculations on the crystalline structure of the monolayer graphene.^69^ The blueshift occurred because of doping in graphene by the charge from the metallic substrate.^44^ With increasing irradiation dosages for both types of samples, it is evident that the C–C bond area is diminishing. The C–O–C, C–OH, and COOH bonds all grow stronger as irradiation doses increase, which suggests that graphene's electrical conductivity declines as doses increase, as suggested by the DFT calculations on the changes of the Dirac point, inter-band formation, band opening, and changes in bond distances at the sites next to the defects. The adsorption of oxygen by graphene causes it to lose electrical conductivity as the radiation exposure increases.^28^ In addition, the XPS peak position changes as a function of irradiation dose confirming that after gamma irradiation, C–C bonds in graphene were broken and other bonds appeared. By combining the experimental characterization of the pristine and defective graphene samples and DFT calculations of molecular and electronic structures, the present study supports the irradiation mechanism of formation of point defects, bond distortion around the defects, and functional group formation as irradiation dose increases.

## Author contributions

C. P. C., A. S., and E. V. conceived the project. C. P. C. performed all the experiments. C. P. C. analysed the data and wrote the manuscript. M. R. G., J. W., G. V., and E. V. provided comments for the manuscript. G. V. and E. V. provided funding. A. S., M. R. G., G. V., and E. V. supervised the project.

## Conflicts of interest

There are no conflicts to declare.

## Supplementary Material

NA-OLF-D5NA00162E-s001

## Data Availability

The data supporting the findings of this study were generated during the experiments; however, they are not publicly available due to U.S. government restrictions and regulatory policies.
