# Supplementary material for: Gamma irradiation effects on physical and electronic properties of monolayer CVD grown graphene
Source: Nanoscale Adv. 2025 Jun 3;7(14):4303–12. doi: 10.1039/d5na00162e (PMC12150860; doi:10.1039/d5na00162e)
Supplement: NA-007-D5NA00162E-s001 [file NA-007-D5NA00162E-s001.pdf]

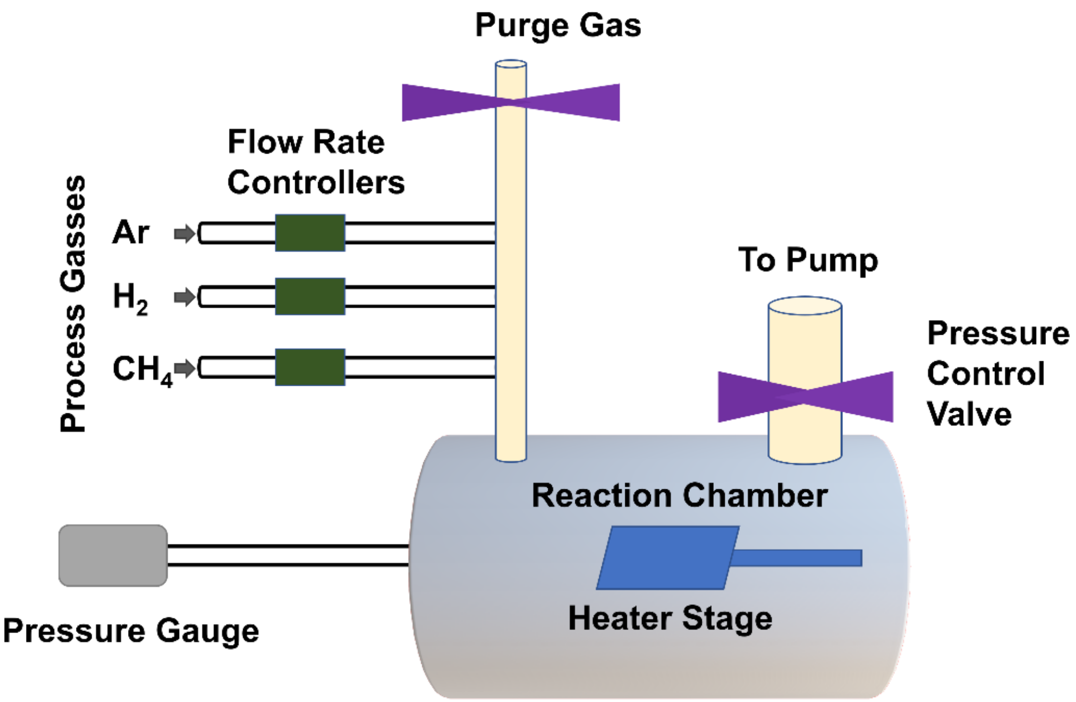

Figure S1: Schematic of gas flow in the graphene nanoCVD reactor.

Table. S1 Electronic properties of pristine and defected monolayer graphene (MLG).

Data of Band Structure and Density of States	Pristine monolayer graphene	1 point defect in graphene	2 point defects in graphene	C-O-C bond with defect in graphene	C-OH bond with defect in graphene	-COOH bond with defect in graphene
E_f (Fermi Energy) (eV)	0.9014	0.6151	0.3890	0.7095	0.7695	1.0334
E_g(Bandgap Energy) (eV)	0.00	0.2778	0.6119	0.8105	0.9527	0.2361
